# Sodium-Glucose Cotransporter-2 (SGLT-2) Inhibitors and Genital Infections in Patients With Diabetic Mellitus and Concomitant Coronary Artery Disease: A Single-Center Experience

**DOI:** 10.7759/cureus.31842

**Published:** 2022-11-23

**Authors:** Bhupesh R Shah, Sanjeev Phatak, Priya Phatak, Harshal B Shah, Isha Phatak, Darshil B Shah

**Affiliations:** 1 Cardiology, Smt. Nathiba Hargovandas Lakhmichand Municipal Medical College, Ahmedabad, IND; 2 Internal Medicine, Vijayratna Diabetes And Diagnostic Treatment Center, Ahmedabad, IND; 3 Diagnostic Radiology, Darshil Diagnostic Center, Ahmedabad, IND; 4 Internal Medicine, Ahmedabad Municipal Corporation Medical Education Trust Hospital, Ahmedabad, IND; 5 Internal Medicine, Smt. Nathiba Hargovandas Lakhmichand Municipal Medical College, Ahmedabad, IND

**Keywords:** genital infection, side effects, coronary artery disease, anti-diabetic drugs, diabetes mellitus

## Abstract

Objective

To investigate the incidence of genital infection due to the use of sodium-glucose cotransporter-2 (SGLT-2) inhibitors in patients with type 2 diabetes mellitus (T2DM) concomitant coronary artery diseases (CAD).

Methods

A single-center, physician-initiated study was conducted at a tertiary-care center in India. The study enrolled patients with T2DM who were taking SGLT-2 inhibitors for at least two months and divided them into two groups: patients with concomitant CAD as the case group and without CAD as the control group. Demographic data and medical history of patients were documented using a standard questionnaire. Itching and swelling were the signs used for the diagnosis of genital infection.

Results

A total of 270 consecutive patients with T2DM were enrolled and divided into two groups: 48 patients with CAD as the case group and 222 patients without CAD as the control group. The mean age of patients with CAD was 63.27±7.53 years and without CAD was 58.32±14.89 years. The mean HbA1C levels were 8.40±1.71% in the case group and 8.60±7.20% in the control group. A total of 14.6% of patients with CAD and 12.6% of patients without CAD were found to have genital infections (p=0.712). SGLT-2 inhibitors were stopped in only six patients who had genital infections and all the patients were managed using anti-fungal cream and via maintenance of proper hygiene. The overall incidence of genital infection was about 12.96%, of which only 2.7% required discontinuation of this crucial therapy.

Conclusion

In conclusion, the incidence of genital infection with the use of SGLT-2 inhibitors is similar among patients with T2DM with concomitant CAD and without CAD. The measures to prevent genital infection should be strongly emphasized. However, larger, well-designed studies are required to validate the current findings.

## Introduction

Type 2 diabetes mellitus (T2DM), a non-communicable disease, is a significant global public health concern that is more prevalent in India. One of the most recent families of anti-diabetic drugs is sodium-glucose cotransporter-2 (SGLT-2) inhibitors (empagliflozin, dapagliflozin, canagliflozin, ipragliflozin, ertugliflozin, sotagliflozin, and luseogliflozin) that have improved the management of diabetes. SGLT-2 inhibitors enhance the excretion of glucose in the urine by inhibiting SGLT-2 transporters, which are responsible for the reabsorption of glucose from the proximal convoluted tubules in the kidney. Patients with T2DM frequently experience cardiovascular complications, and SGLT-2 inhibitors have demonstrated preventive benefits against these in several studies [1-4].

Generally, patients with T2DM are at risk of urinary tract and genital infections due to elevated urinary glucose levels. In addition, the use of SGLT-2 inhibitors, which pharmacologically increases urinary glucose, may further exacerbate the risk of infection. Several studies indicated a possible association between SGLT-2 and genital/urinary tract infections [5,6]. However, the occurrence of genital infection with the use of SGLT-2 inhibitors has not been reported previously in patients with T2DM and concomitant coronary artery disease (CAD). 

Therefore, the present study sought to investigate whether the incidence of genital infections increases with the use of SGLT-2 inhibitors in patients with T2DM and concomitant CAD compared to patients without CAD.

## Materials and methods

This was a single-center, physician-initiated study carried out at a tertiary care center in India, between August 2021, and September 2022. The study included consecutive patients who presented with T2DM and were taking SGLT-2 inhibitors for at least two months and were divided into two groups based on the concomitant presence of CAD as the case group and the absence of CAD as the control group. The study was approved by the institutional ethics committee (S.No./IEC/2021/3274) and written informed consent was obtained from all the enrolled patients.

Demographic data and medical history of patients were documented using a standard questionnaire. Clinical characteristics of all patients were recorded. Genital infection was documented if the enrolled patients reported itching as a symptom and swelling/redness as a sign in the genital area. Glycated hemoglobin (HbA1c) levels were also tested and documented.

Statistical analysis was performed using SPSS Statistics v. 20 (IBM Corp., Armonk, NY). Continuous variables were expressed as mean and standard deviations, and categorical variables were stated as frequency count and percentage. A chi-square test was used to study categorical variables and an independent sample t-test was used to compare the continuous variables. A p-value of <0.05 was considered statistically significant.

## Results

A total of 270 consecutive patients with T2DM were enrolled in our study and were divided into two groups based on the concomitant presence of CAD: 48 patients with CAD as the case group and 222 patients without CAD as the control group. The mean age of patients in the case group was 63.27±7.53 years and in the control group was 58.32±14.89 years. The study reported male dominance with 38 (79.2%) males in the case group and 112 (50.5%) in the control group. Baseline characteristics of all enrolled patients were comparable in terms of chronic kidney disease (0 vs 5 (2.3%); p=0.590) and previous stroke (0 vs 1 (0.5%); p=1). None of the patients in the control group had a history of cardiovascular disease; however, 48 (17.78%) patients in the case group had a history of cardiovascular disease.

The mean HbA1C levels were 8.40±1.71% in the case group and 8.60±7.20% in the control group (p=0.842). Both the groups were taking SGLT-2 inhibitors: dapagliflozin (19 (39.6%) vs 169 (76.1%)), empagliflozin (28 (58.3%) vs 43 (19.4%)), canagliflozin (0 (0.0%) vs 1 (0.5%)), and remogliflozin (1 (2.1%) vs 9 (4.1%)). Detailed baseline and clinical characteristics, and clinical findings of patients in the case and control groups are outlined in Table 1.

**Table 1:** Baseline characteristics and clinical findings CAD: coronary artery disease; BMI: body mass index; UTI: urinary tract infection; SGLT-2: sodium-glucose cotransporter-2; HbA1C: glycated hemoglobin

	Total (n=270)	With CAD (N=48)	Without CAD (N=222)	p-value
Age (years), mean ± SD	59.20±13.98	63.27±7.53	58.32±14.89	0.026
Gender				
Female, n (%)	120 (44.44%)	10 (20.80%)	110 (49.50%)	<0.001
Male, n (%)	150 (55.56%)	38 (79.2%)	112 (50.5%)	
Height (cm), mean ± SD	161.48±10.21	163.71±8.28	161.00±10.54	0.096
Weight (kg), mean ± SD	75.75±11.95	73.96±9.01	76.13±12.47	0.255
BMI (kg/m^2^), mean ± SD	29.18±4.86	27.62±2.94	29.52±5.13	0.014
Diabetes mellitus, n (%)	270 (100.0%)	48 (100.0%)	222 (100.0%)	
Duration of diabetes mellitus				
1-5 years, n (%)	85 (31.48%)	9 (18.8%)	76 (34.2%)	0.016
6-10 years, n (%)	86 (31.85%)	13 (27.1%)	73 (32.9%)	
>10 years, n (%)	99 (36.67%)	26 (54.2%)	73 (32.9%)	
Hypertension, n (%)	186 (68.89%)	43 (89.6%)	143 (64.4%)	0.001
History of cardiovascular disease, n (%)	48 (17.78%)	48 (100.0%)	00	
Chronic kidney disease, n (%)	5 (1.85%)	0 (0.0%)	5 (2.3%)	0.590
Previous stroke, n (%)	1 (0.37%)	0 (0.0%)	1 (0.5%)	1.000
SGLT-2 inhibitor				
Dapagliflozin, n (%)	188 (69.63%)	19 (39.6%)	169 (76.1%)	<0.001
Empagliflozin, n (%)	71 (26.30%)	28 (58.3%)	43 (19.4%)	
Canagliflozin, n (%)	1 (0.37%)	0 (0.0%)	1 (0.5%)	
Remogliflozin, n (%)	10 (3.70%)	1 (2.1%)	9 (4.1%)	
Genital tract infection, n (%)	35 (12.96%)	7 (14.6%)	28 (12.6%)	0.712
Baseline HbA1C level (%)	8.57±6.56	8.40±1.71	8.60±7.20	0.842
<7.0, n (%)	58 (21.48%)	9 (18.75%)	49 (22.07%)	0.340
≥7.0 to 8.0, n (%)	80 (29.63%)	11 (22.92%)	69 (31.08%)	
>8.0, n (%)	132 (48.90%)	28 (58.33%)	104 (46.85%)	

In the present study, a total of seven (14.6%) CAD patients and 28 (12.6%) without CAD reported genital infections and the difference was not statistically significant (p=0.712). Of 35 patients with genital infections in both groups, SGLT-2 inhibitors were stopped in only six patients (17.14%) and all the patients were managed with anti-fungal cream and proper maintenance of hygiene. The overall incidence of genital infection was 12.96% of which only 2.7% (6/270) required discontinuation of this crucial therapy.

## Discussion

The present study sought to compare the occurrence of genital infections among patients with T2DM who received SGLT-2 inhibitor therapy and had concomitant CAD with those without CAD. The key finding emerging from the study was that patients with T2DM who received SGLT-2 inhibitors and had concomitant CAD were associated with a numerically higher incidence of genital infections as compared to those without CAD patients, but the difference was not statistically significant (p=0.712).

Pharmacologically, SGLT-2 inhibitors are responsible for increased concentration of glucose in the urine (renal glucosuria), thus providing a favorable environment for pathogens to thrive, which subsequently results in genital infections [7]. Mechanistically, SGLT-2 inhibitors prevent the reabsorption of glucose by inhibiting the SGLT-2 channels present in proximal convoluted tubules of the kidney, thus promoting its excretion in urine [8].

Pooled safety data from 12 placebo-controlled phase-IIb/III trials (n=4545 diabetic patients) reported a higher incidence of genital infections in the dapagliflozin group (2.5 mg: 4.1%, 5 mg: 5.7%, 10 mg: 4.8%) as compared to the placebo group (0.9%) [9]. Similarly, a double-blind, parallel-group, placebo-controlled study involving 546 adults with T2DM documented a higher incidence of genital infections in the dapagliflozin group (2.5 mg: 8%, 5 mg: 13%, 10 mg: 9%) compared to the placebo group (5%) [10]. Pooled safety data from four randomized, placebo-controlled phase-III trials (n = 2477 patients) demonstrated that patients who are on empagliflozin therapy for T2DM had a higher incidence (10 mg: 4.2%, 25 mg: 3.6%) of genital infections as compared to those on placebo therapy (0.7%) [11]. All these studies cumulatively confirmed that SGLT-2 inhibitors are associated with a substantial risk of urinary tract and genital infections. Multiple studies have demonstrated that females who received SGLT-2 inhibitors were at an increased rate of genital infections compared with those receiving placebos [9,12,13].

SGLT-2 inhibitors have demonstrated cardiovascular protective activity and the desired reduction of cardiovascular and overall mortality. The discovery of SGLT-2 inhibitors has greatly revolutionized the field of diabetes and heart failure as several studies consistently documented their cardioprotective effects [14, 15]. The incidence of genital infections in patients with T2DM who are at high cardiovascular risk is greatly varied. EMPA-REG OUTCOME trial is a randomized, double-blinded, placebo-controlled study that included 7028 adults with T2DM who are at high risk of cardiovascular disease, demonstrated a comparable incidence of genital infections in the 10 mg empagliflozin group with the placebo (18.2% vs. 18.1%) and lower incidence of genital infections in 25 mg empagliflozin group than the placebo group (17.8% vs. 18.1%) [16]. Likewise, the Canagliflozin Cardiovascular Assessment Study (CANVAS) involving a total of 10,142 participants with T2DM and high cardiovascular risk demonstrated a higher incidence of male genital infections in the canagliflozin group compared to the placebo group (34.9% vs. 10.8%) [17]. The DECLARE-TIMI 58 trial was a randomized, double-blinded, multi-national, placebo-controlled, phase-3 trial of dapagliflozin in patients with T2DM and established atherosclerotic cardiovascular disease or multiple risk factors for atherosclerotic cardiovascular disease. The incidence of genital tract infections in the dapagliflozin group was roughly approaching the placebo (1.5% vs 1.6%) [18]. All these data were consistent with the reassuring results of a recent meta-analysis of 86 randomized controlled trials which showed a significant increase in urinary tract infections in patients with the SGLT-2 group compared to the placebo.

To the best of our knowledge, none of the studies have compared the incidence of genital infections among patients with T2DM taking SGLT-2 inhibitors and had concomitant CAD with those without CAD. In the present study, the patients who received SGLT-2 inhibitors for T2DM and had concomitant CAD reported a numerically higher incidence of genital infections as compared to those without CAD but the difference was not statistically significant (p=0.712). In short, the prevalence of genital infections has greatly varied among patients with cardiac or other comorbidities.

This present study has some limitations worth mentioning. It was a single-center study with a small sample size. Also, the difference in the number of patients in the case and control groups was higher due to consecutive enrolment of patients during the given duration of the study. The study also didn’t report the risk ratio which might have given more clear details about the association between SGLT-2 inhibitors and genital infection in CAD patients. Therefore, further larger studies with better design characteristics are warranted to provide a clear picture of the effect of SGLT-2 inhibitors on the incidence of genital infections in patients with T2DM with concomitant CAD.

## Conclusions

In conclusion, the study stated that the incidences of genital infection due to the use of SGLT-2 inhibitors are similar among patients with T2DM and concomitant CAD and without CAD. However, further larger studies on a greater number of patients are required to prove its association and also to validate our findings. Moreover, measures to prevent genital infection should be strongly emphasized in these groups of patients.
